# Effects of posterior lumbar nonfusion surgery with isobar devices versus posterior lumbar interbody fusion surgery on clinical and radiological features in patients with lumbar degenerative diseases: a meta-analysis

**DOI:** 10.1186/s13018-022-03015-6

**Published:** 2022-02-21

**Authors:** Jianbin Guan, Tao Liu, Wenhao Li, He Zhao, Kaitan Yang, Chuanhong Li, Ningning Feng, Guozheng Jiang, Yongdong Yang, Xing Yu

**Affiliations:** Dongzhimen Hospital Affiliated to Beijing University of Chinese Medicine, Beijing, 100700 China

**Keywords:** Isobar device, Lumbar nonfusion surgery, Posterior lumbar interbody fusion, Meta-analysis

## Abstract

**Purpose:**

The aim of this study was to systematically evaluate the efficacy of posterior lumbar isobar nonfusion with isobar devices versus posterior lumbar interbody fusion (PLIF) in the treatment of patients with lumbar degenerative diseases (LDDs).

**Materials and method:**

We performed a literature review and meta-analysis in accordance with the Cochrane methodology. The analysis included a Group Reading Assessment and Diagnostic Evaluation assessments, Jadad Quality Score evaluations, and Risk of Bias in Randomized Studies of Interventions assessments. The PubMed, Ovid, EMBASE, Web of Science, MEDLINE, CNKI, VIP and WanFang databases were searched to collect and compare relevant randomized controlled trials and cohort studies of isobar nonfusion and PLIF in the treatment of lumbar degenerative diseases. The retrieval time was from database inception to June 2021. Two evaluators independently screened the literature, extracted data, and evaluated the quality of the included studies. Outcome measures of interest included low back pain, disability, and radiological features. The protocol for this systematic review was registered on INPLASY (2021110059) and is available in full on inplasy.com (https://inplasy.com/inplasy-2021-11-0059/).

**Results:**

Of the 7 RCTs, 394 patients met the inclusion criteria. The meta-analysis results showed that isobar nonfusion surgery shortened the surgical duration (*P* = 0.03), reducing intraoperative bleeding (*P* = 0.001), retained the ROM of surgical segment (*P* < 0.00001) and the ROM of the lumbar spine (*P* < 0.00001), and reduced the incidence of ASD (*P* = 0.0001). However, no significant difference in the postoperative ODI index (*P* = 0.81), VAS score of LBP (*P* = 0.59, VAS score of lower limb pain (*P* = 0.05, and JOA score (*P* = 0.27) was noted.

**Conclusions:**

Posterior lumbar nonfusion surgery with isobar devices is superior to PLIF in shortening the surgical duration, reducing intraoperative bleeding, retaining the ROM of surgical segments and the lumbar spine to a certain extent, and preventing ASD. Given the possible publication bias, we recommend further large-scale studies.

## Introduction

Lumbar fusion surgery is not only the gold standard therapy for lumbar degenerative diseases (LDDs) after failed conservative nonsurgical treatment for at least six months, but also a classic clinical surgical treatment in spinal surgery [1]. It can effectively relieve the symptoms of nerve root compression, reconstruct the spinal sequence, and strengthen spinal stability. Lumbar interbody fusion changes the normal biomechanical environment of the surgical segment, which causes stress concentration and accelerates the degeneration of adjacent segments. Complications, such as adjacent segment disease (ASD), have received increasing attention [2]. Dynamic fixation and nonfusion techniques provide stability of surgical segments and retain a certain degree of lumbar mobility, which hopefully reduce the increase in compensatory activity and stress of adjacent vertebrae, to reduce the incidence of ASD. The isobar system (Scient,X) is a semirigid pedicle screw fixation system that can provide relatively flexible three-dimensional control and be clinically applied to fusion surgery to stimulate bone growth, shorten the fusion time, dynamically protect segments adjacent to rigid surgically fused segments and nonfusion dynamic fixation to reduce the pressure on intervertebral discs and facets of adjacent segments, and retain the range of motion (ROM) of the surgical segment and the lumbar spine [3]. Recently, many reports on the clinical efficacy of isobar system fusion surgery have been published, and a meta-analysis showed that PLIF with the isobar system has some advantages in improving ODI, JOA and VAS scores compared with traditional titanium rods [4]. However, systematic reports on the clinical efficacy of isobar nonfusion surgery versus traditional posterior lumbar interbody fusion (PLIF) are limited. Therefore, this study collected relevant studies comparing the postoperative efficacy of isobar nonfusion surgery versus PLIF in the treatment of lumbar degenerative diseases, screened the literature according to strict inclusion and exclusion criteria, and extracted relevant data for meta-analysis. We aimed to compare the surgical duration, intraoperative bleeding, preoperative and postoperative low back pain, leg pain and clinical function. Postoperative radiological changes were compared, including surgical segment ROM and lumbar ROM, and we also summarized the number of postoperative ASD cases. By systematically evaluating the efficacy of the two surgical methods, we hope to provide an evidence-based medical basis for the clinical treatment of lumbar degenerative diseases.

## Methods

This meta-analysis was performed in accordance with the Preferred Reporting Items for Systematic Reviews and Meta-Analyses (PRISMA) statement [5] and the Cochrane Handbook [6]. Ethical approval was not required since this is a meta-analysis of published studies.

### Inclusion and exclusion criteria

To qualify for inclusion, studies had to be a randomized controlled trials (RCTs) comparing both isobar nonfusion surgery versus PLIF surgery in the treatment of lumbar degenerative diseases and clinical and radiological outcomes directly that differed only in surgical methods. The subjects included patients with lumbar degenerative diseases, including lumbar instability, lumbar disc herniation, lumbar spondylolisthesis, lumbar spinal stenosis, etc. The patients had obvious symptoms of low back pain, which were diagnosed by CT or MRI, and had been treated conservatively for at least 6 months. Studies of individuals who underwent procedures involving other instruments (e.g. Dynesys, N-Flex, an interspinous device, and/or GRAF) were not eligible. Biomechanical studies, single-arm studies, literature reviews, case reports, dissertations and conference summaries were also eligible.

### Search strategy

Referring to the search strategy of the Cochrane assistance network, we searched the PubMed, Ovid, EMBASE, Web of Science, MEDLINE, China National Knowledge Internet (CNKI), VIP and Wanfang databases from inception to June 2021. The subject words, free words, and combinations of the two were used. The following search terms were used: “isobar device”, “isobar TTL”, “isobar semirigid device”, “isobar dynamic stabilization device”, “lumbar degeneration”, “nonfusion”, “lumbar dynamic fixation”, “isobar dynamic fixation”, “isobar TTL dynamic fixation”, and “spinal nonfusion surgery” with the Boolean operators AND or OR. At the same time, we traced the references of the included literature, and the meta-analysis related to this research, screened, and evaluated the references to determine potentially eligible articles.

### Literature screening and data extraction

Two researchers conducted the literature search; and strictly followed the inclusion and exclusion criteria for the preliminary screening and secondary screening of the literature. After screening, two independent researchers extracted data from the literature that met the requirements and then the data were reviewed by a third researcher. Regarding any differences in the included literature, a consensus was reached through discussion among all researchers. The missing data in the literature were obtained by contacting the authors by e-mail. The main data extracted in this study included the name of the first author, year of publication, sample sizes of the experimental group and the control group, sex ratio of patients, average age, intervention methods, surgery for lesions, language, follow-up time, and outcome indicators. The extracted data were reviewed to ensure accuracy.

### Quality assessment

This study used the Cochrane risk bias tool [7] for quality evaluation. This tool includes evaluations in seven aspects: random sequence generation, allocation hiding, blinding of participants and implementers, blinding of outcome evaluators, incomplete outcome data, selective reporting, and other biases. The risk of bias in each area was judged as low, high, or unclear. The quality of the studies was evaluated by two researchers.

### Data analysis

All statistical tests were performed using Review Manager 5.3 software (The Cochrane Collaboration), and the results were represented by a forest map. Heterogeneity tests were conducted during data consolidation. If no obvious heterogeneity was identified among the data (*I*^2^ ≤ 50%), a fixed-effects model was used to consolidate the data. When heterogeneity was observed (*I*^2^ > 50%), a random-effects model was used. If the heterogeneity could not be eliminated, a random-effects model was used for descriptive analysis of obvious clinical heterogeneity. The measurement data are expressed as the mean difference (MD) and its 95% confidence interval (CI); odds ratio (or) was used as the efficacy analysis statistic. All tests were 2-sided, and any p value less than 0.05 was deemed significant. We assessed publication bias by visual inspection of funnel plots.

## Results

### Search results and quality evaluation

**Fig. 1:**
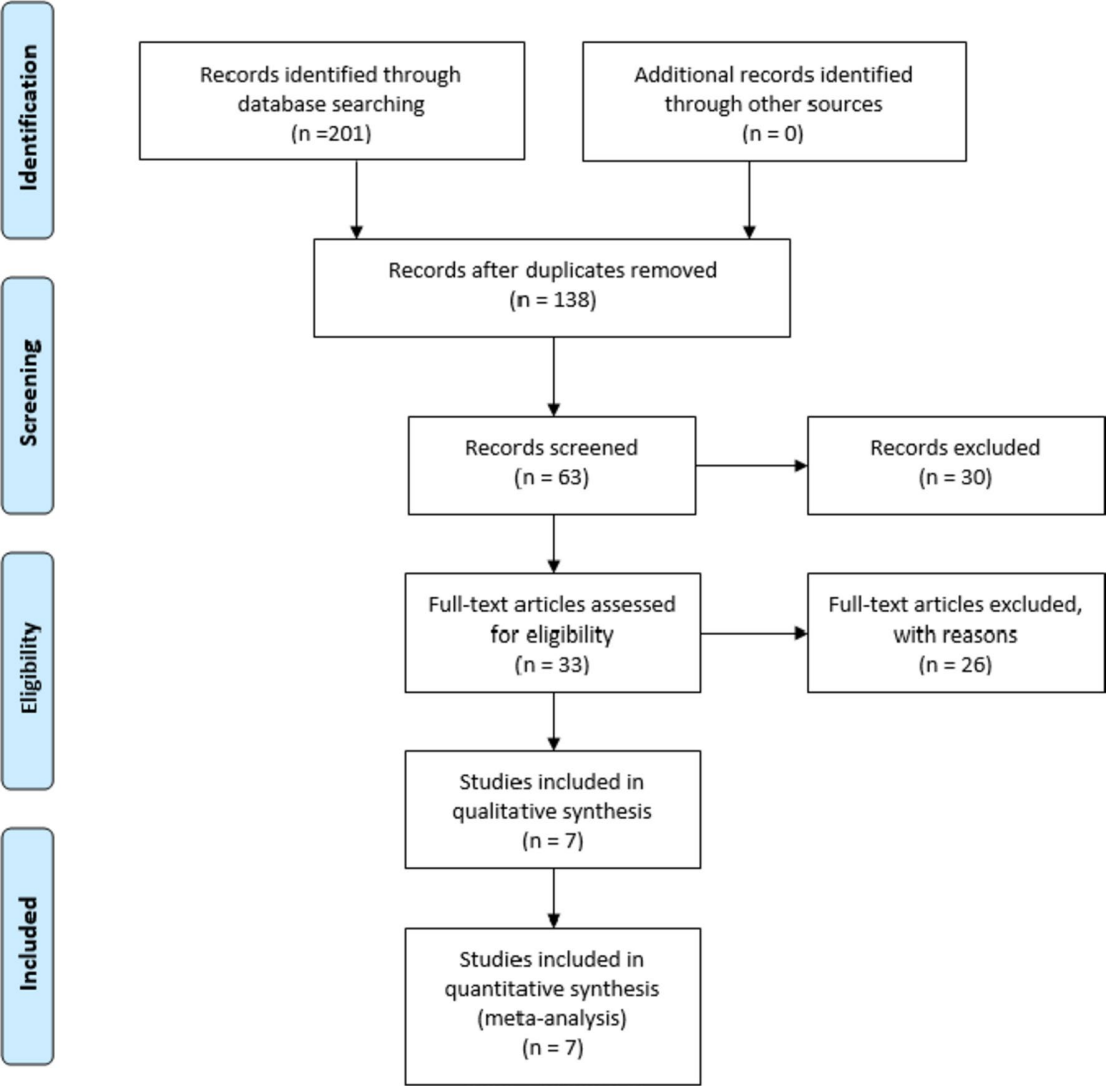
Flow diagram of the study selection process

A total of 201 relevant studies were obtained through a preliminary search, including 66 from PubMed, 12 from Ovid, 13 from EMBASE, 10 from Web of Science, 10 from MEDLINE, 46 from CNKI, 24 from VIP and 68 from WanFang. After eliminating duplicate studies, reading topics and abstracts and full-text re screening, 7 RCT studies [8–14] with 394 patients were finally included. The literature screening flow diagram is shown in Fig. 1, and the basic characteristics of the included studies are shown in Table 1.

**Table 1:** Basic characteristics of the studies included

Inclusion study	Language	Nationality	Segmental operation of lesion	Number of cases		Gender (male/female)		Age (years)		Follow-up time (months)	Outcome indicators
				Isobar	PLIF	Isobar	PLIF	Isobar	PLIF		
Wen 2011 [9]	Chinese	China	1–2	36	36	21/15	14/22	52.3 ± 12.2	51.2 ± 10.9	24	1.2.4.6.9
Li 2011 [10]	Chinese	China	1	14	14	6/8	7/7	48.9 ± 13.6	52.5 ± 12.1	24	1.2.3.4.5.7.8
Xu 2013 [11]	Chinese	China	1	20	20	–	–	–	–	12, 24, 36	3.4.8.9
Liu 2015 [12]	Chinese	China	1–2	20	20	11/9	12/8	53.4 ± 13.2	55.2 ± 8.9	3, 6, 12, 24	3.4.8
Yang 2015 [8]	English	China	1	62	51	36/26	29/22	44.31 ± 13.01	46.59 ± 12.06	6, 24, 48	1.2.3.4.5.7.9
Feng 2017 [13]	Chinese	China	1–2	30	30	12/18	11/19	58.3 ± 7.8	57.6 ± 8.2	6, 12	1.2.4.6.9
Ji 2020 [14]	English	China	2	20	21	8/12	11/10	64 ± 7.78	61 ± 6.5	24, 36	1.2.3.4.7.8.9

^*^Outcome indicators: 1. surgical duration, 2. intraoperative bleeding, 3. Oswestry disability index (ODI), 4.low back pain VAS, 5. lower limb pain VAS, 6. Japanese Orthopaedic Association (JOA), 7. surgical segment ROM, 8. lumbar ROM, 9. the number of postoperative ASD

The specific databases searched, and the number of documents retrieved from each database are as follows: PubMed (*n* = 66), Ovid (*n* = 12), EMBASE (*n* = 13), web of science (*n* = 10), MEDLINE (*n* = 10), CNKI (*n* = 46), VIP (*n* = 24), WanFang Data (*n* = 68).

### Quality assessment

**Fig. 2:**
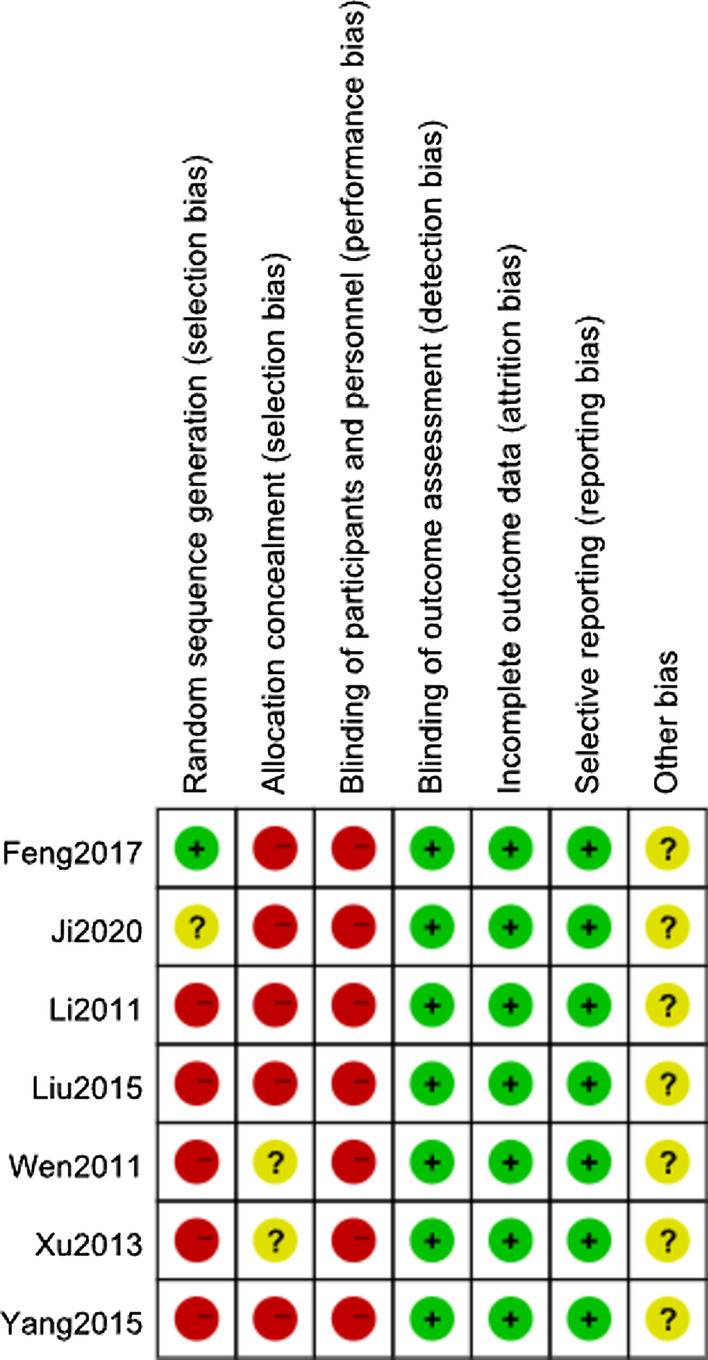
Risk of bias summary. Plus sign indicates low risk of bias. Minus sign indicates high risk of bias, and question mark bias unclear

Due to the particularity of surgical treatment and ethical requirements, the patients’ right to know and personal will must be fully respected when grouping, so neither the patient nor the surgeon can implement blinding. Therefore, the included three studies were high risk in terms of randomization, allocation concealment, and blinding of participants and personnel. None of the seven studies withdrew or was lost to follow-up, and the data were complete. Quality assessment is shown in Fig. 2.

### Meta-analysis results

#### Comparison of the surgical duration for isobar nonfusion and PLIF

**Fig. 3:**
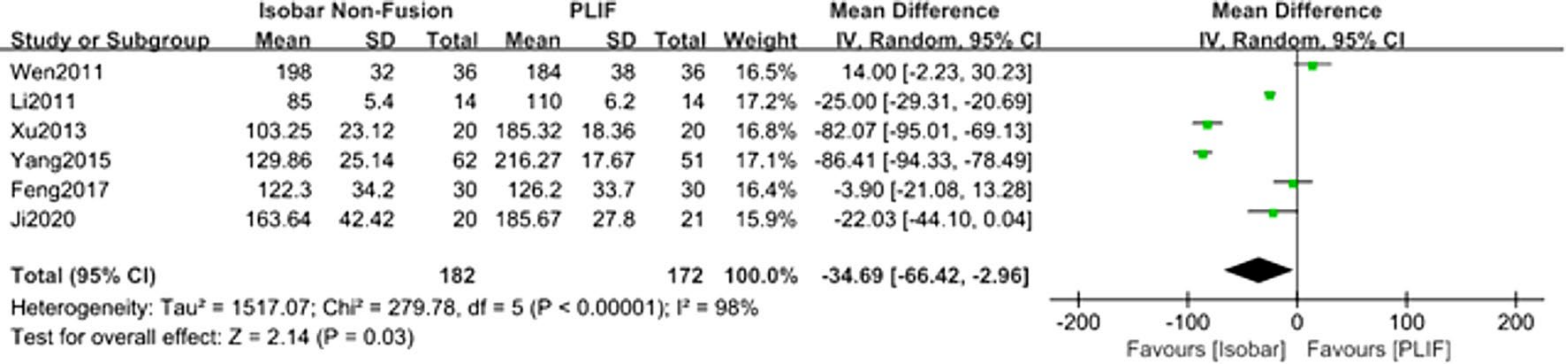
Forest plot of comparison: surgical duration between isobar nonfusion and PLIF

Six studies [8–11, 13, 14] reported the surgical duration and intraoperative bleeding of the two methods. The results of meta-analysis of random effect model showed that there was significant difference in surgical duration between isobar group and PLIF Group [MD = − 34.69; 95% CI: (− 66.42, − 2.96); *P* = 0.03]. (Table 2, Fig. 3).

**Table 2:** Summary of meta-analysis results

Outcome indicators	Follow-up time (month)	Included number	Number of cases		Heterogeneity detection		Effect model	Meta-analysis results	
			ISOBAR	PLIF	*I*^2^ (%)	*P*		MD/OR (95% CI)	*P*
Surgical duration [8–11, 13, 14]	–	6	162	152	98	< 0.00001	Random	− 34.69 (− 66.42, − 2.96)	0.03
Intraoperative bleeding [8–11, 13, 14]	–	6	162	152	98	< 0.00001	Random	− 124.57(− 200.48, − 48.65)	0.001
ODI [8, 10–12, 14]	24	5	130	123	0	0.56	Fixed	− 0.11(− 1.05, 0.82)	0.81
VAS for LBP [8–14]	12	7	198	189	0	0.52	Fixed	0.05 (− 0.14, 0.25)	0.59
VAS for lower limb pain [8, 10]	24	2	72	62	35	0.21	Fixed	− 0.29 (− 0.56, − 0.01)	0.05
JOA score [9, 13]	12	2	66	66	0	0.84	Fixed	0.38 (− 0.29, 1.06)	0.27
Surgical segment ROM [8, 10, 14]	24	3	92	83	97	< 0.00001	Random	7.75 (7.35, 8.16)	< 0.00001
Lumbar ROM [10–12, 14]	24	4	94	75	0	0.75	Fixed	7.95 (6.37, 9.54)	< 0.00001
Number of ASD case [8, 9, 11, 13, 14]	12	5	164	155	0	0.96	Fixed	0.14 (0.04, 0.46)	0.0001

#### Comparison of the intraoperative bleeding for isobar nonfusion and PLIF

**Fig. 4:**
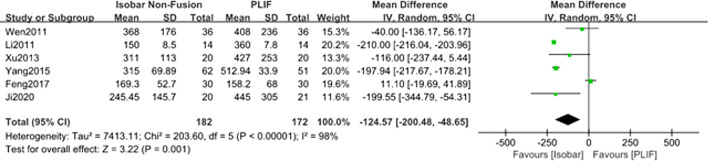
Forest plot of comparison: intraoperative bleeding between isobar nonfusion and PLIF

Six studies [8–11, 13, 14] reported the intraoperative bleeding of the two methods during the operation. The results of meta-analysis of random effect model showed that there was a significant difference in the intraoperative bleeding between isobar group and PLIF Group [MD = − 124.57; 95% CI (− 200.48, − 48.65); *P* = 0.001] (Table 2, Fig. 4).

#### Comparison of the Oswestry Disability Index (ODI) scores for isobar nonfusion and PLIF

**Fig. 5:**
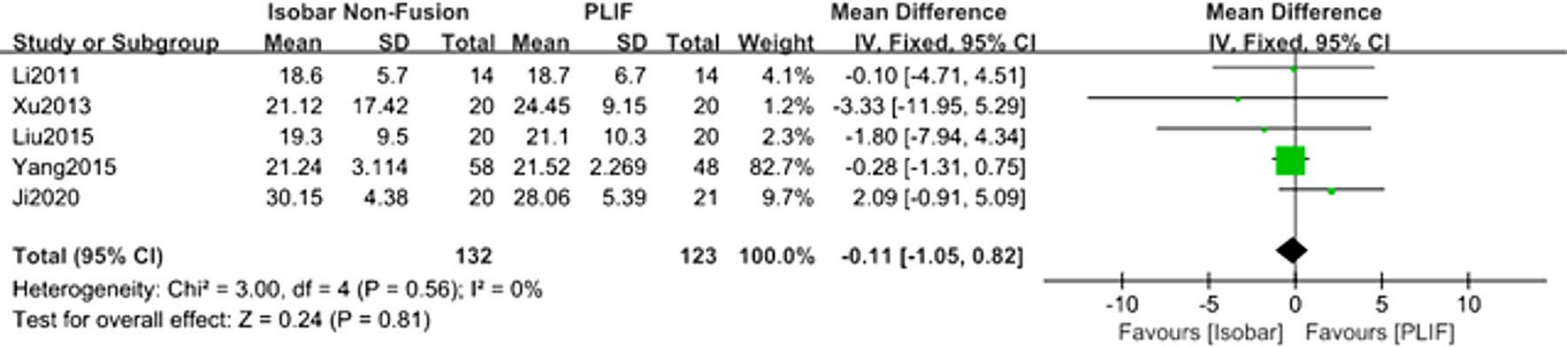
Forest plot of comparison: ODI scores between isobar nonfusion and PLIF

Five studies [8, 10–12, 14] reported the changes of ODI after approximately 24 months of follow-up. The meta-analysis results of fixed effect model showed that there was no significant difference between isobar group and PLIF group in improving postoperative ODI [MD = − 0.11; 95% CI (− 1.05, 0.82); *P* = 0.81] (Table 2, Fig. 5).

#### Comparison of the visual analogue scale (VAS) scores for low back pain (LBP) for isobar nonfusion and PLIF

**Fig. 6:**
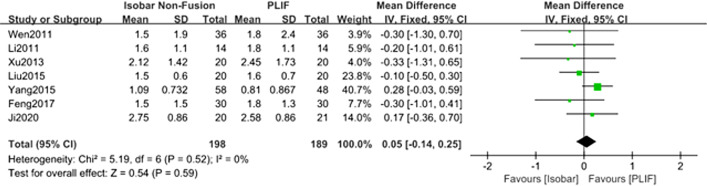
Forest plot of comparison: VAS scores for LBP between isobar nonfusion and PLIF

Seven studies [8–14] reported the compared VAS scores of postoperative of low back pain after approximately 12 months of follow-up. The meta-analysis results of fixed effect model showed that there was no significant difference between isobar group and PLIF group in improving vas of postoperative low back pain [MD = 0.05; 95% CI (− 0.14, 0.25); *P* = 0.59].(Table 2, Fig. 6).

#### Comparison of the visual analogue scale (VAS) scores for lower limb pain for isobar nonfusion and PLIF

**Fig. 7:**
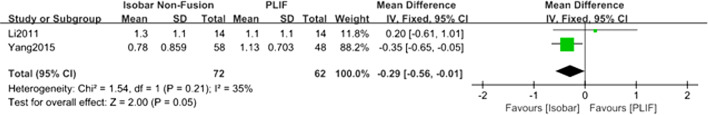
Forest plot of comparison: VAS scores for lower limb pain between isobar nonfusion and PLIF

Two studies [8, 10] reported the changes of vas in lower limb pain after approximately 24 months of follow-up. The meta-analysis results of fixed effect model showed that there was no significant difference between isobar group and PLIF group in improving vas in lower limb pain [MD = − 0.29; 95% CI (− 0.59, − 0.01); *P* = 0.05] (Table 2, Fig. 7).

#### Comparison of the Japanese Orthopaedic Association (JOA) scores for isobar nonfusion and PLIF

**Fig. 8:**
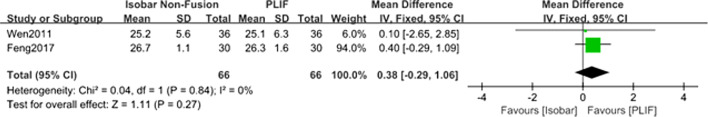
Forest plot of comparison: JOA scores between isobar nonfusion and PLIF

Two studies [9, 13] reported the improvement of JOA after approximately 12 months of follow-up. The fixed effect model meta-analysis showed that there was no significant difference between isobar group and PLIF group in improving postoperative JOA [MD = 0.38; 95% CI (− 0.29, 1.06); *P* = 0.27] (Table 2, Fig. 8).

#### Comparison of the changes of surgical segment ROM for isobar nonfusion and PLIF

**Fig. 9:**
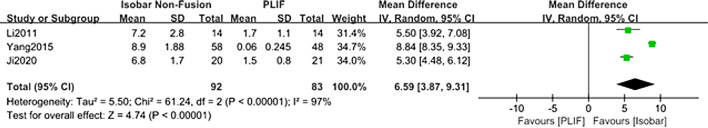
Forest plot of comparison: the changes of surgical segment ROM between ISOBAR NONFUSION and PLIF

Three studies [8, 10, 14] on imaging indexes reported the changes of surgical segment ROM after approximately 24 months of follow-up. The meta-analysis results of fixed effect model showed that the retention of surgical segment ROM in isobar group was significantly higher than that in PLIF group, and the difference between the two groups was statistically significant [MD = 7.75; 95% CI (7.35, 8.16); *P* < 0.00001] (Table 2, Fig. 9).

#### Comparison of the changes of lumbar ROM for isobar nonfusion and PLIF

**Fig. 10:**
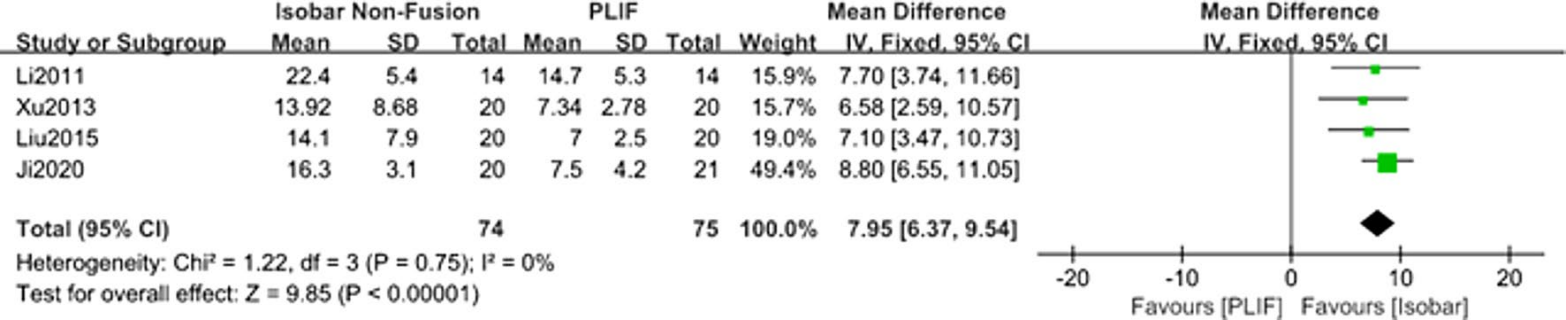
Forest plot of comparison: the changes of lumbar ROM between isobar nonfusion and PLIF

Four studies [10–12, 14] reported the changes of lumbar ROM after approximately 24 months of follow-up. The meta-analysis results of fixed effect model showed that the postoperative lumbar ROM in isobar group was significantly greater than that in PLIF group, and the difference between the two groups was statistically significant [MD = 7.95; 95% CI (6.37, 9.54); *P* < 0.00001] (Table 2, Fig. 10).

#### Comparison of the number of postoperative ASD case for isobar nonfusion and PLIF

**Fig. 11:**
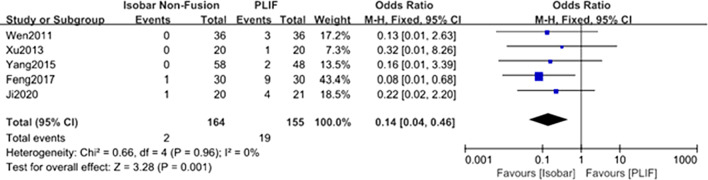
Forest plot of comparison: the incidence of ASD case between isobar nonfusion and PLIF

Five studies [8, 9, 11, 13, 14] reported the number of postoperative ASD case after two types of operation after approximately 12 months of follow-up. The results of meta-analysis of fixed effect model showed that the number of postoperative ASD case after isobar nonfusion was significantly higher than PLIF [OR 0.14; 95% CI (0.01, 0.46); *P* = 0.0001] (Table 2, Fig. 11).

### Publication bias

**Fig. 12:**
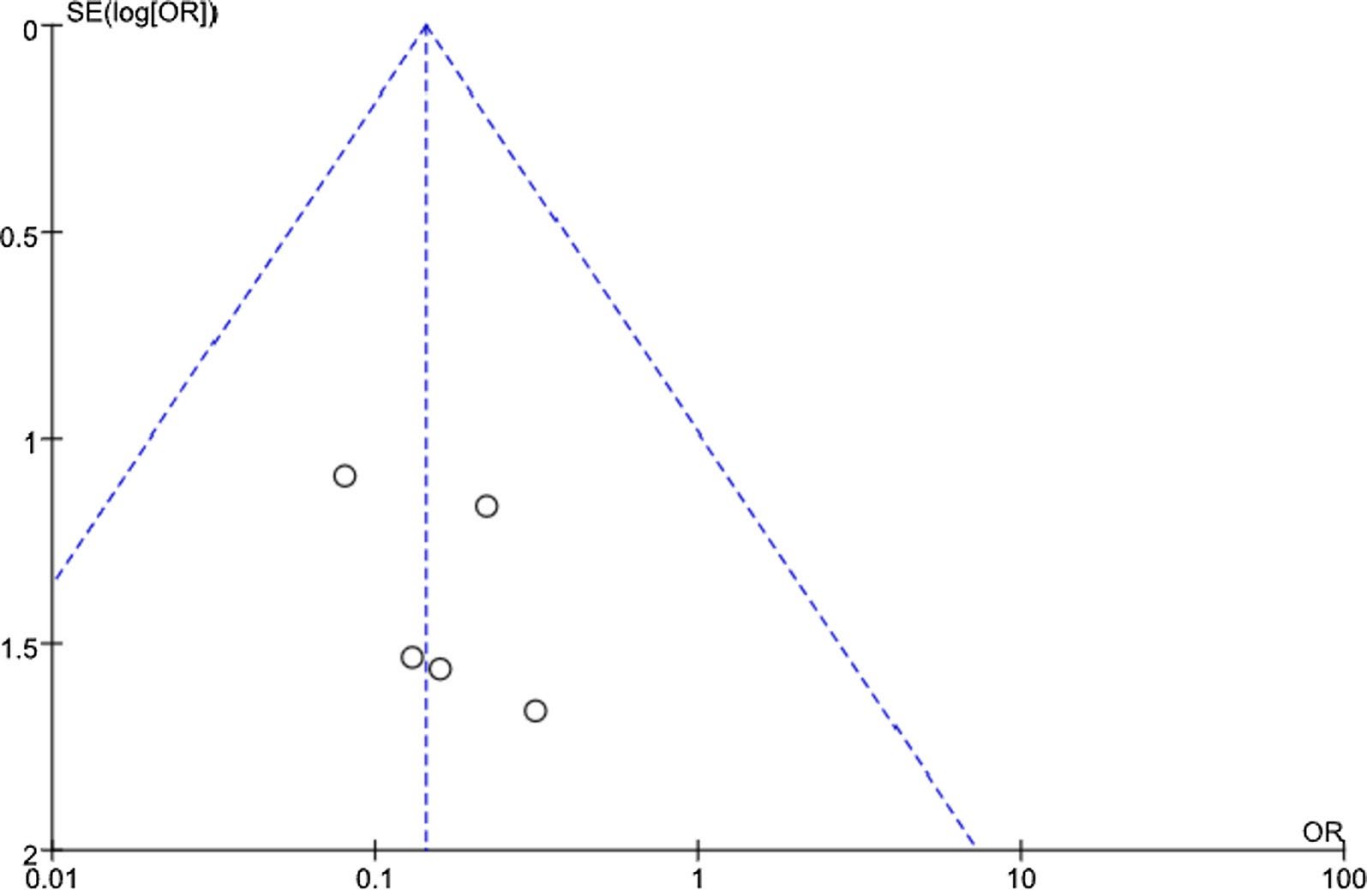
Funnel plot to detect publication bias for the studies

The number of postoperative ASD case is the common outcome index of five studies [8, 9, 11, 13, 14], and it is also the main indicator for evaluating the two groups. Therefore, this outcome index was used to make a funnel plot to detect publication bias, as shown in Fig. 12. Visual inspection of the funnel plots showed symmetry, suggesting that there was no publication bias.

### Sensitivity analysis

The heterogeneity of surgical duration (*I*^2^ = 98%), intraoperative bleeding (*I*^2^ = 98%) and surgical segment ROM (*I*^2^ = 97%) is high. The included literature is excluded one by one, and the remaining literature is combined to show high heterogeneity, indicating that the results of this meta-analysis are reliable, and the heterogeneity may be related to operation technology of surgeons, postoperative nursing measures in the hospital and the psychological character of patients.

### GRADE evidence quality evaluation

The intraoperative bleeding, lumbar ROM, and the number of ASD case were high-level evidence quality. Surgical duration, ODI, VAS score for LBP and surgical segment Rom were moderate-level evidence quality. Vas score for LBP and JOA score were low-level evidence quality. It indicates that this result still needs to be further confirmed by higher quality RCTs, as shown in Table 3.

**Table 3:** Results of GRADE evidence evaluation

Outcome indicators	Risk of bias	Inconsistency	Indirectness	Imprecision	Publication bias	Upgrade conditions	Overall quality of evidence	Importance
Surgical duration	Serious^a^	Serious^b^	Not serious	Not serious	Undetected	None	⊕⊕⊕ Moderate	Important
Intraoperative bleeding	Serious^a^	Serious^b^	Not serious	Not serious	Undetected	None	⊕⊕⊕ Moderate	Important
ODI	Serious^a^	Not serious	Not serious	Not serious	Undetected	None	⊕⊕⊕⊕ High	Important
VAS for LBP	Serious^a^	Not serious	Not serious	Not serious	Undetected	None	⊕⊕⊕⊕ High	Important
VAS for lower limb pain	Serious^a^	Not serious	Not serious	Serious^c^	Undetected	None	⊕⊕ Low	Not important
JOA score	Serious^a^	Not serious	Not serious	Serious^c^	Undetected	None	⊕⊕ Low	No Important
Surgical segment ROM	Serious^a^	Serious^b^	Not serious	Serious^c^	Undetected	None	⊕⊕⊕ Moderate	Important
Lumbar ROM	Serious^a^	Not serious	Not serious	Not serious	Undetected	None	⊕⊕⊕⊕ High	Important
Number of ASD case	Serious^a^	Not serious	Not serious	Not serious	Undetected	None	⊕⊕⊕⊕ High	Important

^a^Random grouping, allocation concealment and blinding cannot be implemented

^b^The heterogeneity test showed that there was high heterogeneity between the studies

^c^Small number of studies

## Discussion

Since Albee and Hibbs used spinal fusion surgery to treat spinal deformity in 1911, spinal fusion surgery has been widely used in the treatment of LDDS. PLIF, as a classic operation for the treatment of lumbar degenerative diseases, can effectively relieve nerve roots compression and strengthen the stability of the lumbar spine, but it causes some complications, such as accelerated degeneration of adjacent segments of the intervertebral disc, decreased lumbar mobility, chronic LBP, and lower limb pain [15]. Currently, as clinicians have deepened their understanding of degeneration of the adjacent segment of the fusion zone and ASD, they have realized that the change in the stress transmission mode of the diseased lumbar segment rather than the magnitude of the stress is an important factor in the LBP caused by degeneration of the lumbar spine [16]. Therefore, we infer that LBP is not always proportional to the degree of lumbar degeneration. Isobar devices were first developed from isolock abd were designed by French Albert Aiby in 1993. The concept is to obtain physiological fusion by interbody bone graft fusion and rigid fixation with a pedicle screw system at the fusion segment. This device is used at the upper adjacent vertebral segment of the fusion segment, to form a dynamic “transition zone” between the fusion segment and the normal segment, which is conducive to recovering lumbar lordosis and restoring normal mechanical conduction to prevent or slow the incidence of ASD. *Gilles Perrin* applied the device to the fixation and fusion of spondylolysis segments for the first time [17]. During the operation, the fixation device was placed in the upper adjacent segments to prevent ASD. The retrospective study with an average follow-up of 8.27 years obtained satisfactory results. Among them, the fusion rate of the surgical segments was 95%, while the compensatory activity of the adjacent segments was shared by the fixation device. Only one case had obvious ASD after the operation, and no revision cases due to internal fixation failure were reported. Subsequently, the device was improved by Scient,X Company in 1997 and renamed the TTL system. The system is composed of a titanium rod with a diameter of 5.5 mm and a microjoint. A microjoint is composed of a titanium ring with strong wear resistance, which can provide ± 0.2 mm axial displacement and ± 2° flexion and extension activity. The rod is a prebending rod with an 8° forward flexion angle, which is in line with the normal stress transmission and mechanical environment between vertebral bodies. However, the activity retained by the TTL system is too small, and controversy about whether it can prevent ASD remains [18]. Since the clinical application of isobar nonfusion surgery, many scholars have reported its clinical efficacy. In terms of surgical details, isobar nonfusion surgery can effectively shorten the surgical duration and reduce the intraoperative bleeding compared with PLIF. This view has been widely recognized, and this approach also yields high patient satisfaction [18–22]. However, ongoing debates concern improving postoperative lumbar function, alleviating pain symptoms, and preserving surgical segment ROM and lumbar ROM [23]. Nevertheless, no meta-analysis has explored the efficacy of isobar nonfusion surgery and PLIF in the treatment of lumbar degenerative diseases.

The results of the meta-analysis of operation showed that isobar nonfusion surgery had statistical advantages over PLIF in shortening the surgical duration and reducing intraoperative bleeding, which was also the same as the conclusion of some retrospective cohort studies. The main reasons for our results may be as follows. First, the overall operation steps of PLIF are cumbersome. During the operation, the cartilage must be completely removed to expose the endplate. At the same time, bone grafting and interbody fusion cage need to be placed. However, isobar nonfusion surgery does not require bone grafting and fusion. Only the free detached nucleus pulposus tissue was explored and removed, and the inclusive and prominent intervertebral disc that did not compress the nerve root was retained. The intervertebral space of the operative segment was preserved as much as possible, and the isobar device did not require the metal connecting rod used in the PLIF operation; it can be fixed only by placing a PDS and titanium rod with a microjoint, which is easy to install. Second, a large amount of bleeding occurs during PLIF, which is related to the long operative time of PLIF, and the cartilage plate must be completely removed during the operation, which will also cause blood seepage between vertebral bodies. Considering the high heterogeneity and possible publication bias of the included studies, we tried to contact the corresponding authors of these studies to obtain more detailed data for subgroup meta-analysis, but we did not receive a reply. Therefore, we analysed the heterogeneity, which may be related to differences in surgeons’ surgical proficiency and surgical methods (such as stripping the paravertebral muscle, and treatment of the intervertebral space).

Postoperative clinical function ODI, JOA and VAS scores are important indices to evaluate postoperative pain, functional recovery, and surgical efficacy [24, 25]. Tian et al. [26] conducted a short-term follow-up of more than 1 year for 20 patients with single-segment lumbar degenerative diseases who were dynamically fixed with the TTL system after decompression. The results showed that the JOA score and ODI index of the lumbar spine were significantly improved. Li et al. [27] used an isobar system to treat 37 patients with lumbar stenosis, disc herniation and instability. During the 24-month follow-up, the ODI and VAS scores changed most significantly at 3 months after the operation. Then, the ODI tended to stabilize, and the VAS score had improved significantly at the last follow-up. These studies show that the application of the isobar system in nonfusion surgery can significantly improve postoperative lumbar function and reduce pain symptoms. At present, most studies have shown that applying isobar TTL in nonfusion surgery can significantly improve postoperative lumbar function and reduce pain symptoms, but whether this technique can achieve the same or an even better effect than traditional PLIF is uncertain. The meta-analysis results of this study show no significant difference in ODI, JOA and VAS scores between isobar nonfusion and PLIF at the same follow-up time, indicating that isobar nonfusion can improve postoperative lumbar function and reduce pain similar to PLIF, but the findings do not reflect its advantages in this regard. The main reasons for our results may be as follows. First, low back pain relief and lumbar function recovery mainly depend on complete decompression of the affected nerve roots, but not on the fusion of the responsible segments. Second, the follow-up time included in the study was short (12 months), and medium- and long-term follow-ups may better reflect the advantages of nonfusion.

Imaging indices, and postoperative ASD cases, postoperative surgical segment ROM, adjacent segment ROM and lumbar ROM are important factors for disease recurrence and ASD development. They are also the main research focus with respect to the clinical efficacy of isobar nonfusion surgery. Zhang et al. [28] found that isobar nonfusion surgery can retain ROM in the surgical segment to a certain extent, prevent an increase in compensatory ROM in adjacent vertebrae, and maintain the normal mechanical environment of the lumbar spine. Gao et al. [29] obtained the same results and found that after isobar fixation, the compensatory ROM of adjacent segments decreased significantly compared with that in the PLIF group. However, some scholars have reached different conclusions through follow-ups. Fu et al. [30] performed single-segment nonfusion surgery in 36 patients, and the Pfirrmann level of the intervertebral disc increased 2 years after the operation, in the surgical segments or adjacent segments. Therefore, they concluded that isobar nonfusion surgery cannot effectively prevent the occurrence of ASD. A retrospective study [31] of 20 patients with lumbar degenerative diseases, which also found that isobar nonfusion surgery had no significant effect on the ROM of adjacent segments, and two cases of ASD were noted. The results of a retrospective analysis by Li et al. also showed that during 2-year follow-up, 14 patients suffered ASD, 3 of whom required reoperation [26]. Some scholars who disagree with this view believe that degeneration of adjacent segments is the result of the natural development of lumbar diseases and cannot be simply attributed to nonfusion [32, 33]. Regarding the influence of lumbar ROM, 22 patients had lumbar degenerative diseases for one year, and Qian et al. had no significant difference between the ROM (3.46 ± 1.02) ° of the preoperative surgical segment and the ROM (2.25 ± 0.79)° after the operation, indicating that isobar nonfusion can retain the activity of the surgical segment to a certain extent [34].

Although the research included in this meta-analysis lacks clinical RCTs for ROM analysis of adjacent segments, the two indices of postoperative surgical segment ROM and lumbar ROM can reflect the normal biomechanical structure of the spine. Therefore, these two indices can reflect the protective effect on adjacent vertebrae. Meta-analysis showed that isobar nonfusion surgery could effectively preserve the surgical segments and lumbar ROM compared with PLIF. Theoretically, retaining both activities may delay degeneration of adjacent vertebral bodies. We analysed the possible reasons as follows. First, the elastic coefficient of the isobar system is similar to that of the normal spine, the biomechanical environment of the reconstructed spine is close to normal, normal stress conduction is restored, and degeneration of the intervertebral disc is prevented [35–37]. Moreover, isobar nonfusion surgery can eliminate the abnormal range of motion of surgical segments, while the microjoint of the isobar retains part of the ROM of the surgical segment, to reduce the increase in the compensatory ROM of adjacent segments, with no significant change in lumbar ROM [38].

Regarding postoperative ASD prevention, the results of this meta-analysis showed a significant difference between isobar nonfusion surgery and PLIF after approximately 12 months of follow-up. This study shows that the incidence of ASD after isobar nonfusion (1.22%) is significantly lower than that of PLIF (12.26%), indicating that isobar nonfusion can effectively prevent the occurrence of ASD in the short term. The reasons may be as follows. First, the activity of surgical segments is retained, which reduces the compensatory activity of adjacent segments is reduced. Second, the ROM of the lumbar spine is retained, and patients can carry out a wider range of rehabilitation training after surgery, which facilitates muscles recoverys. Strong muscle function is conducive to reducing the pressure between the lumbar vertebrae and effectively preventing ASD.

### Limitations of this study

Our meta-analysis has the following limitations. First, published and unpublished manuscripts were not considered for inclusion when searching the literature. Second, the number of included studies was small, the number of cases was small, the follow-up time was short, and no subgroup analysis was performed, resulting in a confounding bias of the results. Moreover, other factors affecting the choice of clinical operation methods were not considered, such as operation costs, and disease severity. Third, the ROM of the lumbar spine may change significantly with an increase in surgical segments. Due to the lack of comparison between single-segment and multisegmental isobar nonfusion in the included studies, we did not study the effect of surgical segments on the prognoses of the two groups. Finally, the systematic evaluation included only the surgery duration, intraoperative bleeding, VAS scores for lower limb and low back pain, the ODI, the ROM of postoperative surgical segment and the lumbar spine, and the incidence of postoperative ASD. More imaging indices failed to be systematically evaluated due to the lack of original data.

## Conclusion

Current evidence shows that isobar nonfusion and PLIF have the same effect in improving postoperative VAS, ODI and JOA scores. However, isobar nonfusion has obvious advantages over PLIF in shortening the surgery duration and reducing intraoperative bleeding. At the same time, isobar nonfusion can effectively preserve the ROM of the surgical segment and lumbar spine, which is of great significance for patients’ postoperative functional exercise and daily life. As a mature lumbar nonfusion technology, isobar nonfusion has achieved satisfactory clinical efficacy in short-term follow-ups within 2 years, but randomized controlled trials for spanning more than 2 years are still lacking. Therefore, the medium- and long-term efficacy of this operation must be further confirmed by more high-quality clinical studies.

## Data Availability

All data generated or analysed during this study are included in this published article and its supplementary information files.

## Abbreviations

LDDs: Lumbar degenerative diseases
PLIF: Posterior lumbar interbody fusion
RCT: Randomized controlled trials
MD: Mean differences
OR: Odds ratios
CI: Confidence intervals
JOA: Japanese Orthopaedic Association
VAS: Visual Analogue Scale
LBP: Low back pain
ODI: Oswestry Disability Index
ASD: Adjacent segment degeneration
PRISMA: Preferred Reporting Items for Systematic Reviews and Meta-Analyses
ROM: Range of motion
CNKI: China National Knowledge Infrastructure
